# Supplementary material for: Coevolution of female fidelity and male help in populations with alternative reproductive tactics
Source: Proc Biol Sci. 2021 Jan 27;288(1943):20202371. doi: 10.1098/rspb.2020.2371 (PMC7893278; doi:10.1098/rspb.2020.2371)
Supplement: Supplementary Information [file rspb20202371supp1.docx]

Supplementary Materials

1. The overlapping generations model: supplementary figures of the fitness landscape and the equilibrium frequency of sneakers at different combinations of female fidelity $u$ and male help $h$ values.


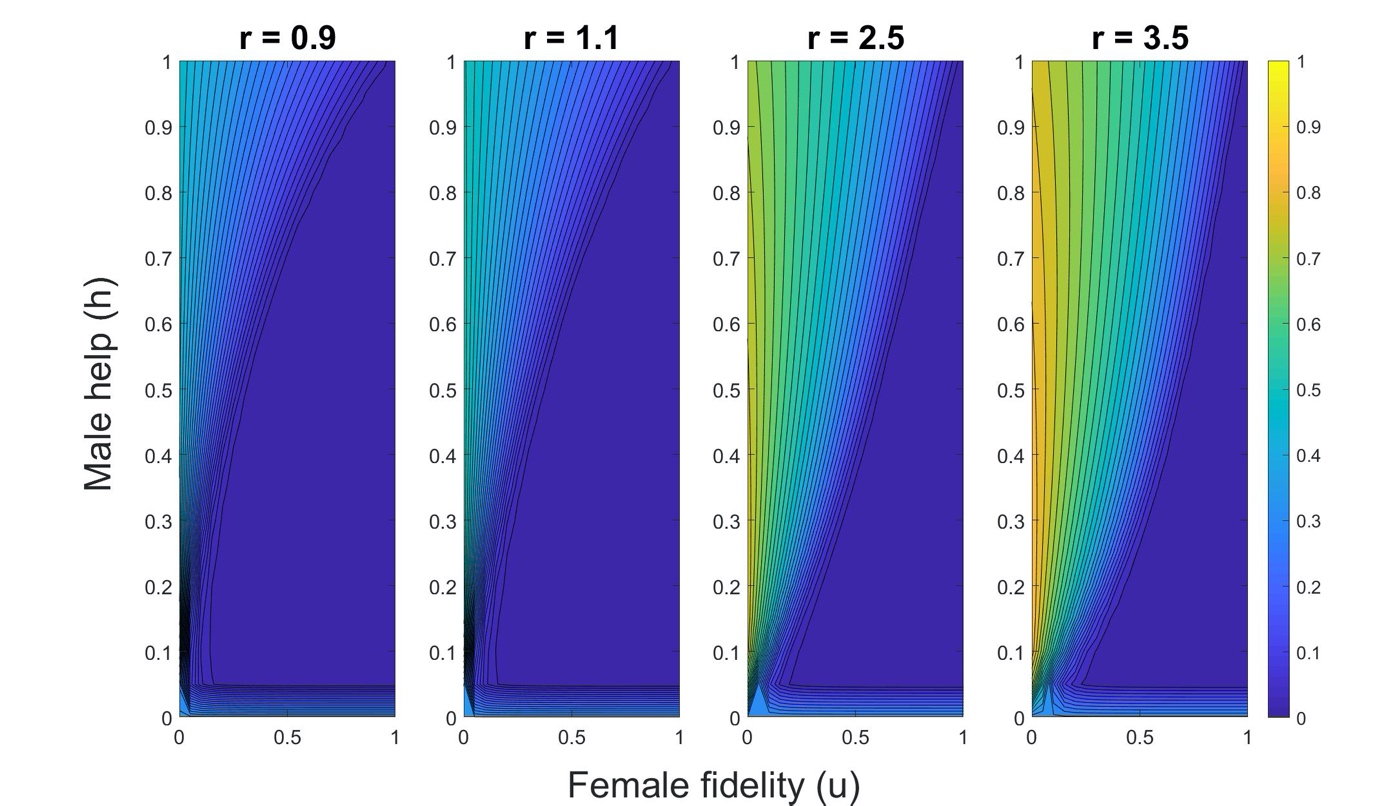


Figure S1. Equilibrium frequency of sneakers in the population at different levels of female fidelity and male help combinations. The parameters used for the numerical solutions correspond to Figure 1 in the main text, with the efficiency of mate guarding $\delta=0.5$.


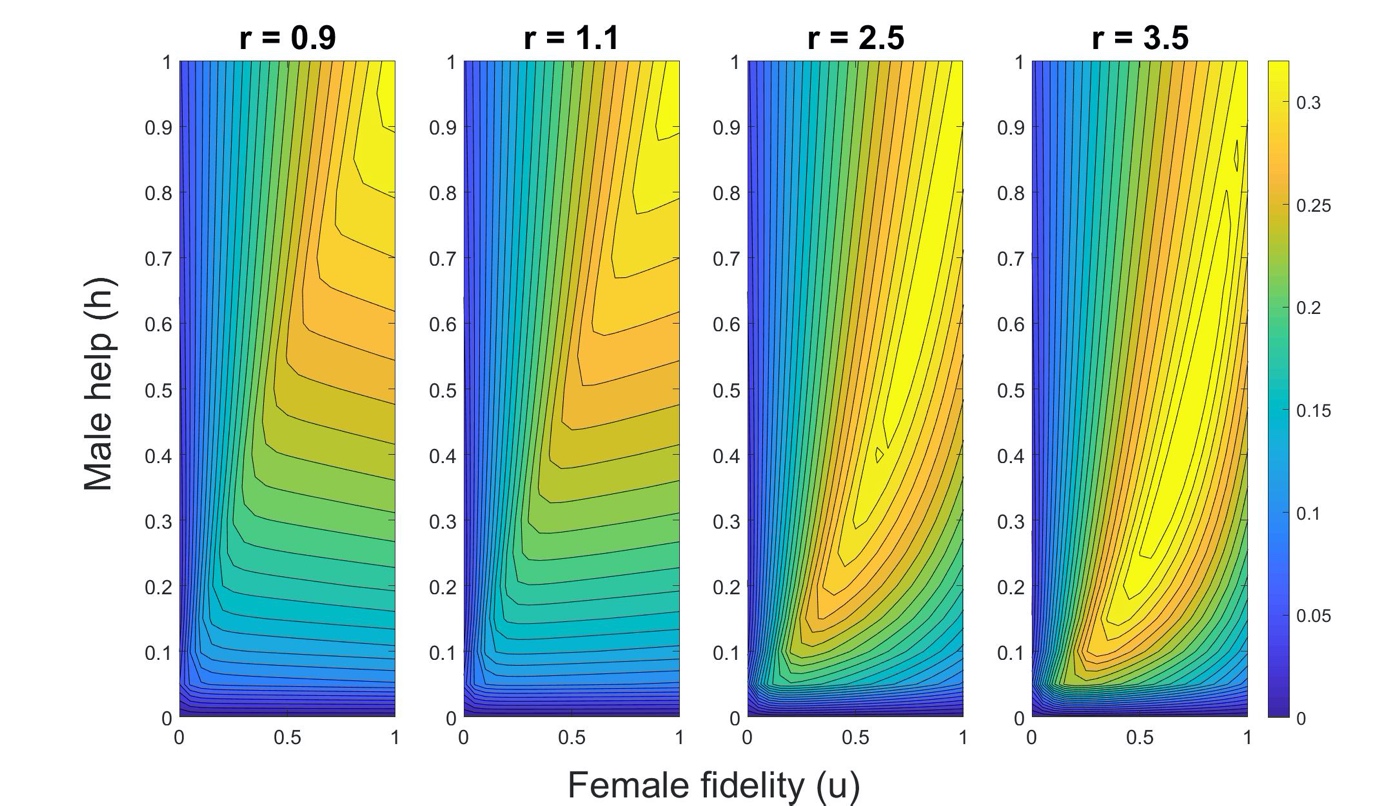


Figure S2. The fitness landscape, represented by the per capita growth rate of the population at evolutionary equilibrium, under different $r$ (survival rate of extra-pair offspring relative to their within-pair siblings) at different levels of female fidelity and male help combinations. The efficiency of mate guarding $\delta=0.1$.


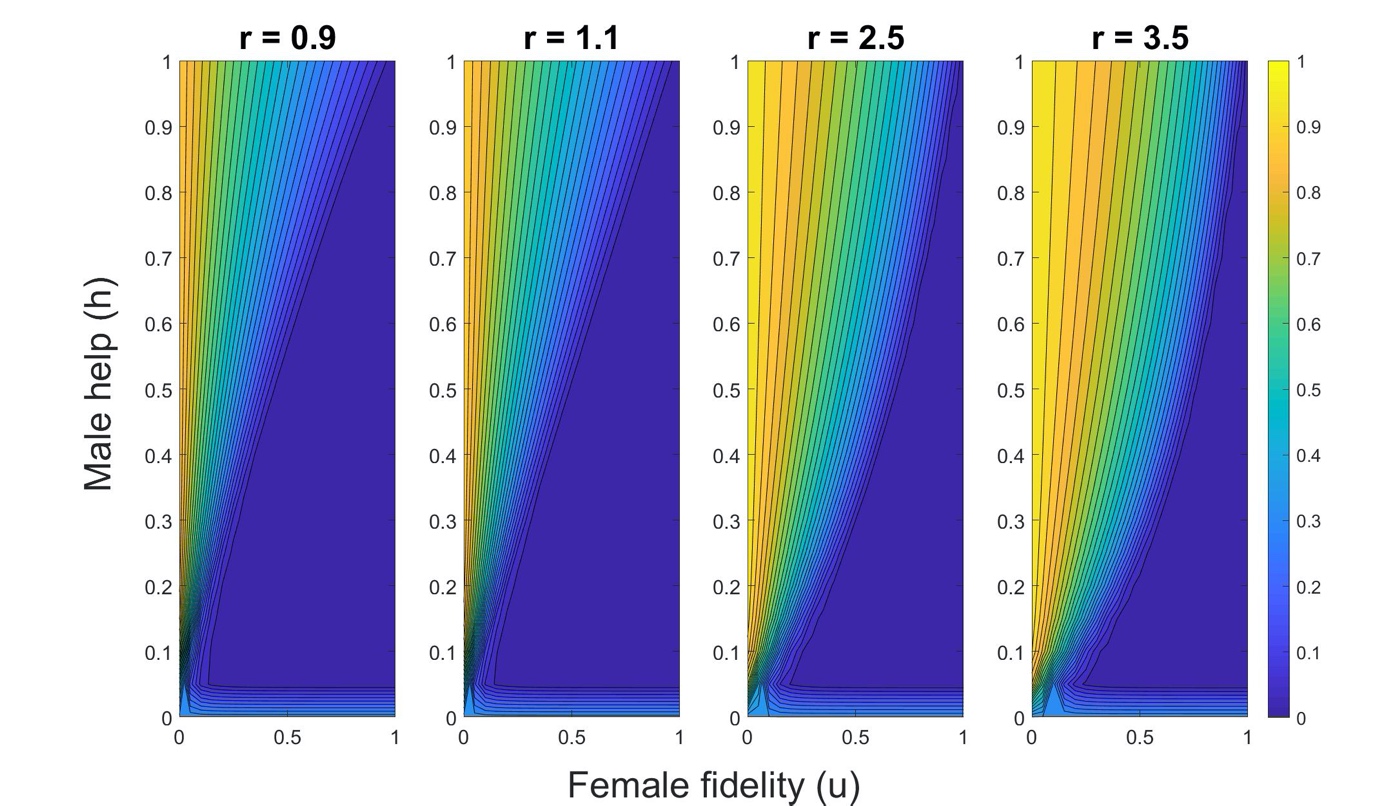


Figure S3. Equilibrium frequency of sneakers in the population at different levels of female fidelity and male help combinations. The parameters used for the numerical solutions correspond to Figure S2, with the efficiency of mate guarding $\delta=0.1$.


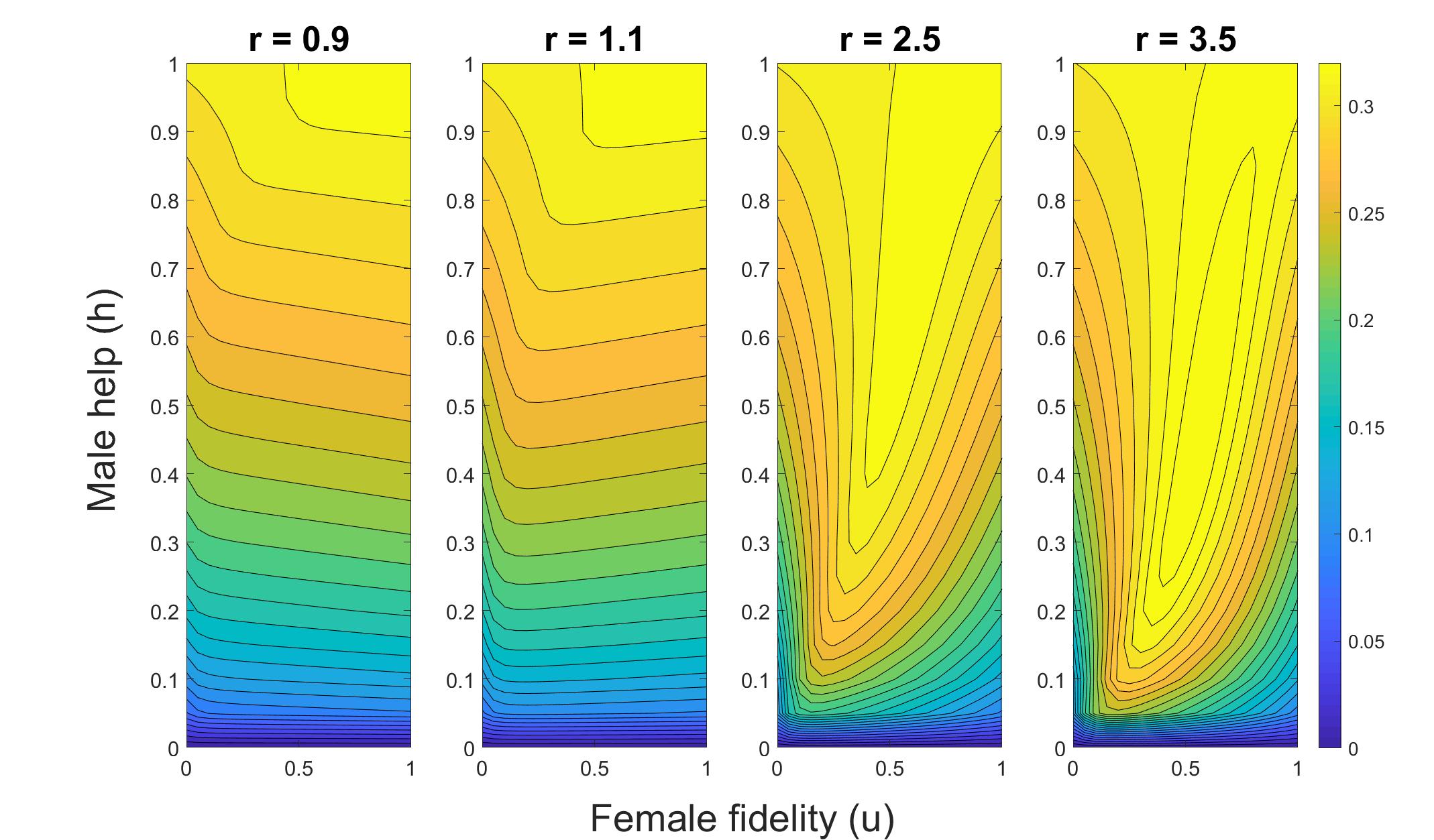
 Figure S4. The fitness landscape, represented by the per capita growth rate of the population at evolutionary equilibrium, under different $r$ (survival rate of extra-pair offspring relative to their within-pair siblings) at different levels of female fidelity and male help combinations. The efficiency of mate guarding $\delta=0.9$.


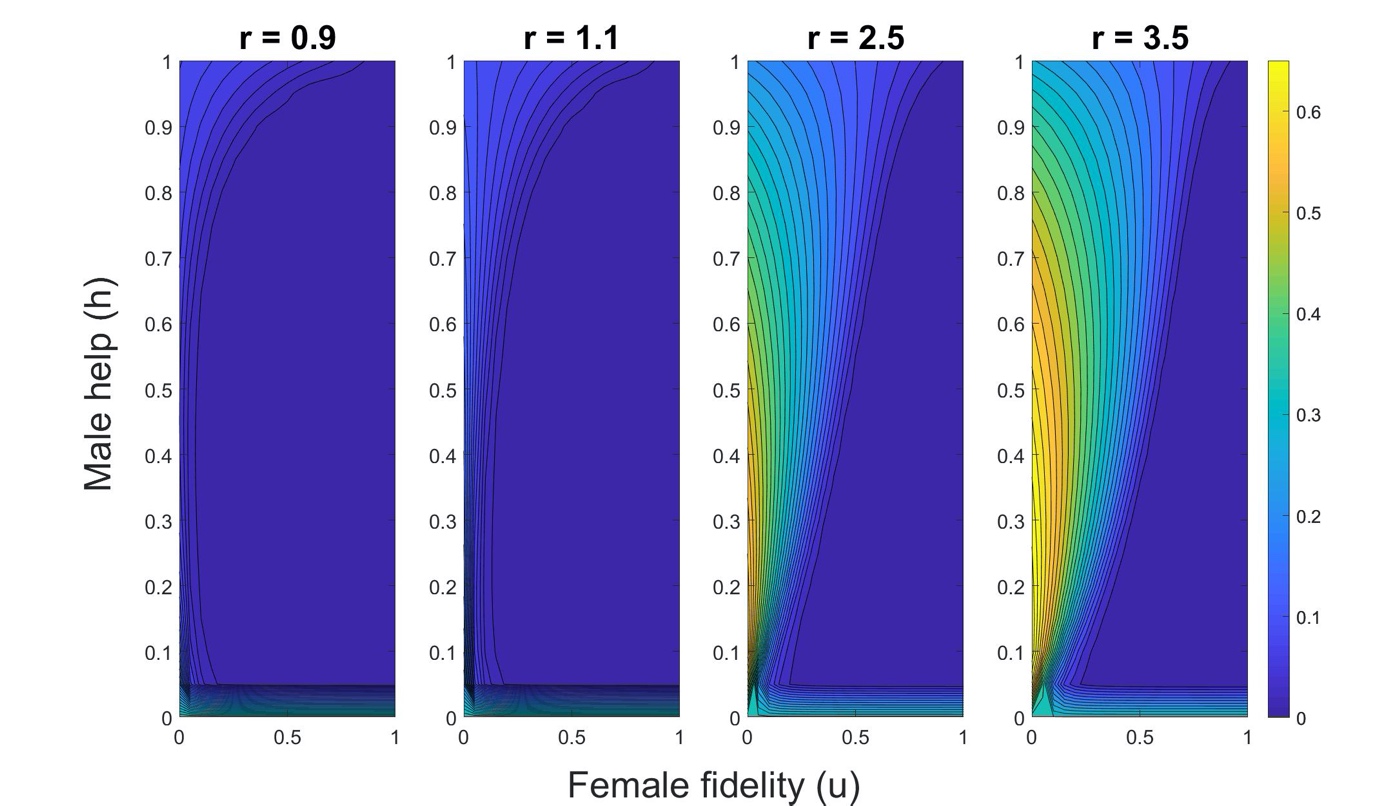


Figure S5. Equilibrium frequency of sneakers in the population at different levels of female fidelity and male help combinations. The parameters used for the numerical solutions correspond to Figure S4, with the efficiency of mate guarding $\delta=0.9$.

1. The non-overlapping generation model: additional description and supplementary figures of the fitness landscape and the equilibrium frequency of sneakers at different combinations of female fidelity $u$ and male help $h$ values.

In the case of non-overlapping populations, the modelling framework largely remains the same as in the case of overlapping population (described in the main text). The main difference between the two models lies in the way of how the increments in the population numbers are described. For the non-overlapping generation model the total number of individuals $N(t)$ at the end of generation $t$ is

| $N(t)=\sum\left( \Delta_{\mathrm{wp}}F_{i}\left( t \right)+\Delta_{\mathrm{wp}}M_{i}\left( t \right)+\Delta_{\mathrm{ep}}F_{i}\left( t \right)+\Delta_{\mathrm{ep}}M_{i}\left( t \right) \right)$, $i=AA, Aa, aa$ |  |
| --- | --- |

where the meaning of $\Delta_{k}F_{i}\left( t \right)$ and $\Delta_{k}M_{i}\left( t \right)$ (*k*=1,2) is the same as in the model for overlapping generations (see the main text). In other words, we assume that the parents die shortly after the reproduction and by the end of each year the population only contains individuals born in the same year. The number of females at the start of generation $t+1$ is given by

| $F_{i}\left( t+1 \right)=\Delta_{\mathrm{wp}}F_{i}\left( t \right)+\Delta_{\mathrm{ep}}F_{i}\left( t \right),$ for $N(t)<N_{0}$; |  |
| --- | --- |
| $F_{i}\left( t+1 \right)=\left( \Delta_{\mathrm{wp}}F_{i}\left( t \right)+\Delta_{\mathrm{ep}}F_{i}\left( t \right) \right)N_{0}/N(t),$ for $N(t)>N_{0}$, |  |

where the number *N_0_* has the meaning of the carrying capacity. The dynamics for males in the population can be obtained in similar ways.

When female fecundity $R$ is sufficiently high (survival of the population with non-overlapping populations generally requires higher values of *R*). As, in the main text the per capita population growth rate *W_t_* within a generation is given by expression (7), which we plot for large *t*. The parametric diagrams for the per capita population growth rate as well as the proportion of sneakers in the population are shown below constructed for the same parameter values as in the main text.


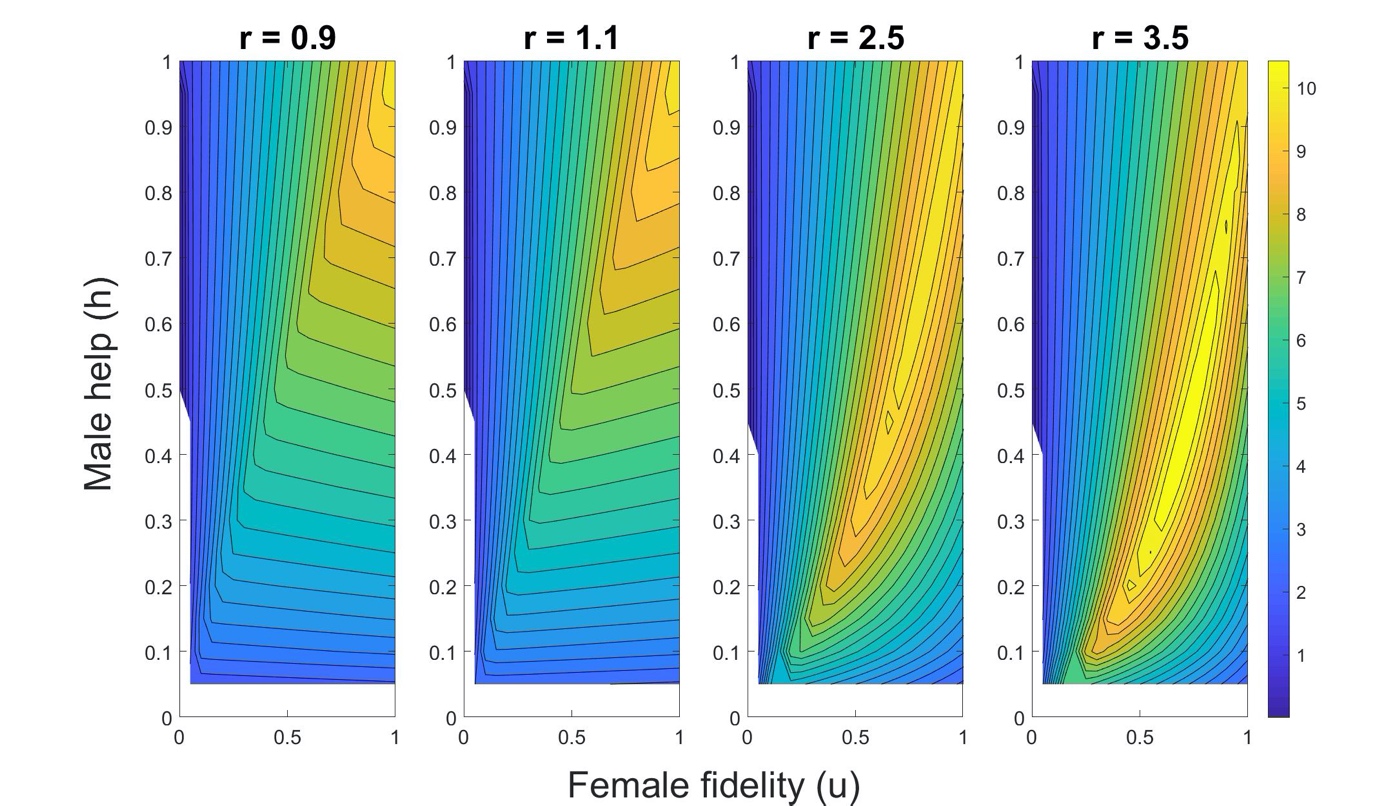


Figure S6. The fitness landscape under the non-overlapping generation model, represented by the per capita growth rate of the population at evolutionary equilibrium, under different $r$ (survival rate of extra-pair offspring relative to their within-pair siblings) at different levels of female fidelity and male help combinations. The efficiency of mate guarding $\delta=0.1$. The empty parameter region at low values or female fidelity and/or male help is due to population extinction caused by low survival rate of the offspring.


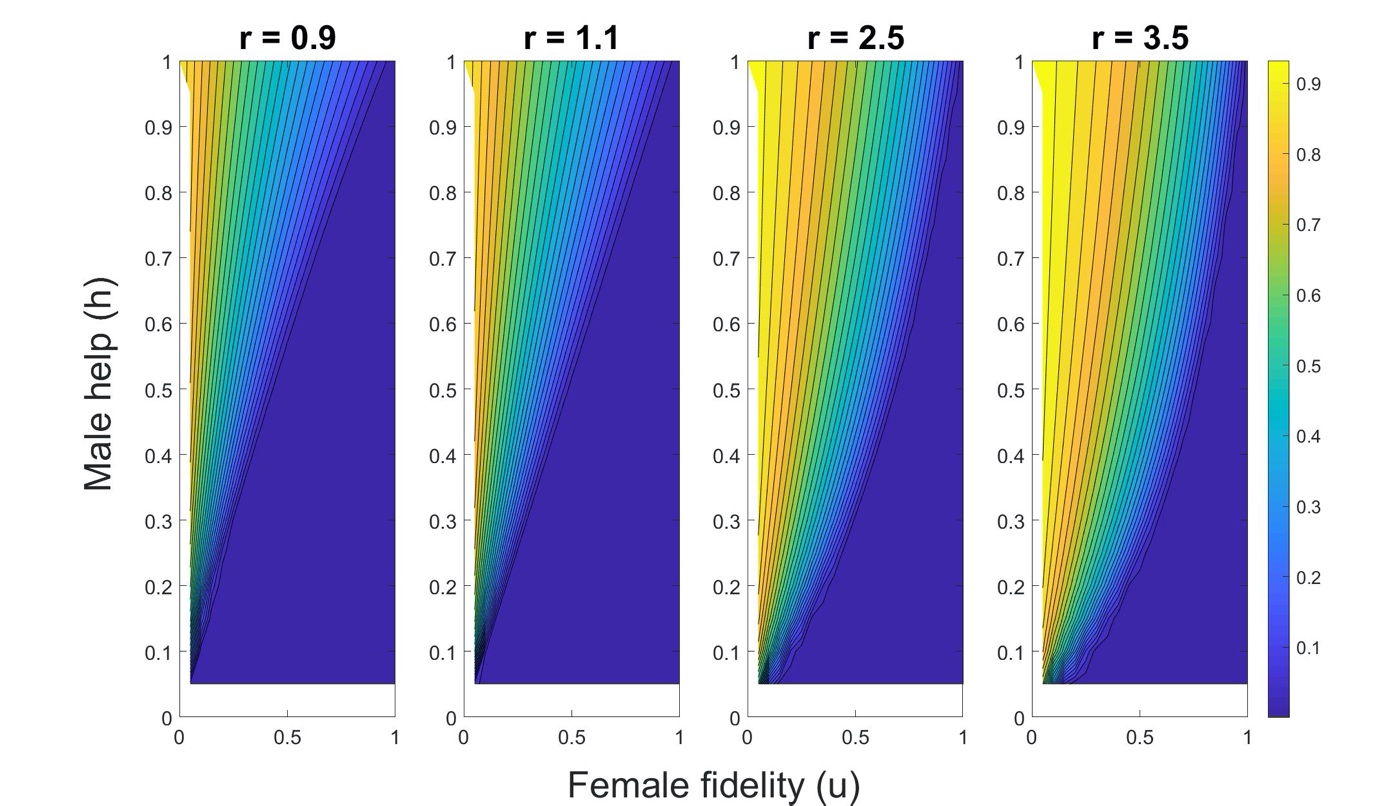


Figure S7. Equilibrium frequency of sneakers in the population under the non-overlapping generation model at different levels of female fidelity and male help combinations. The parameters used for the numerical solutions correspond to Figure S6, with the efficiency of mate guarding $\delta=0.1$.


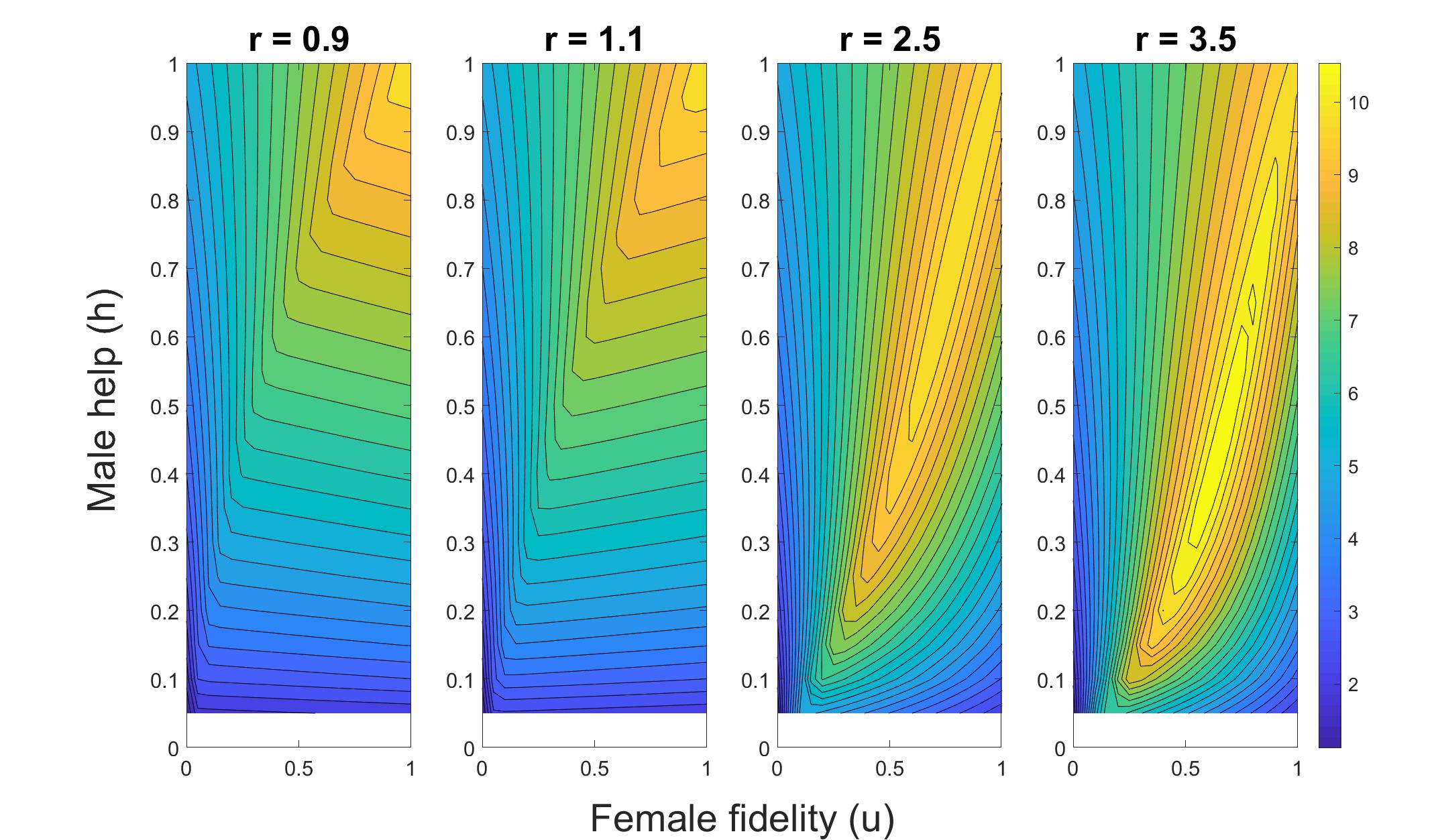


Figure S8. The fitness landscape under the non-overlapping generation model, represented by the per capita growth rate of the population at evolutionary equilibrium, under different $r$ (survival rate of extra-pair offspring relative to their within-pair siblings) at different levels of female fidelity and male help combinations. The efficiency of mate guarding $\delta=0.5$. The empty parameter region at low values or female fidelity and/or male help is due to population extinction caused by low survival rate of the offspring.


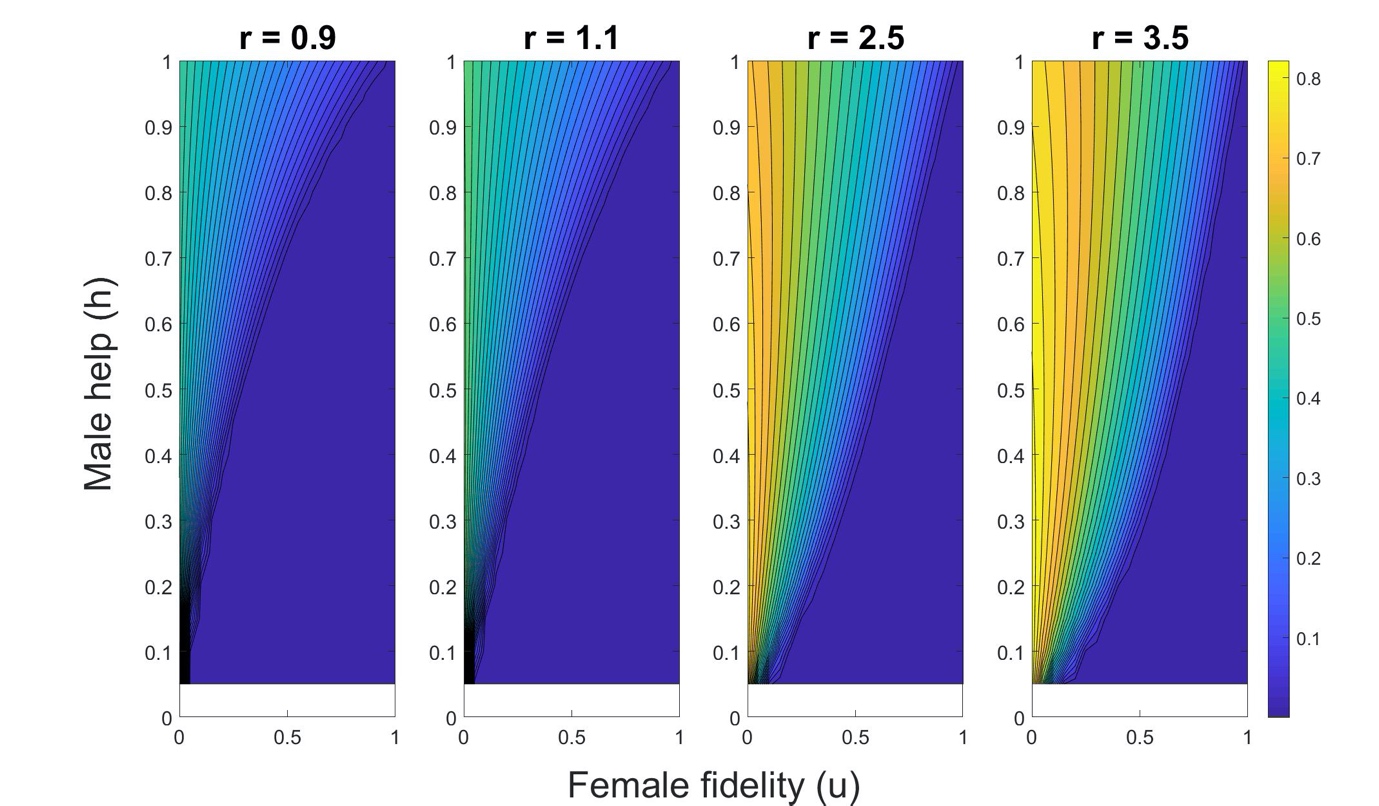


Figure S9. Equilibrium frequency of sneakers in the population under the non-overlapping generation model at different levels of female fidelity and male help combinations. The parameters used for the numerical solutions correspond to Figure S8, with the efficiency of mate guarding $\delta=0.5$.


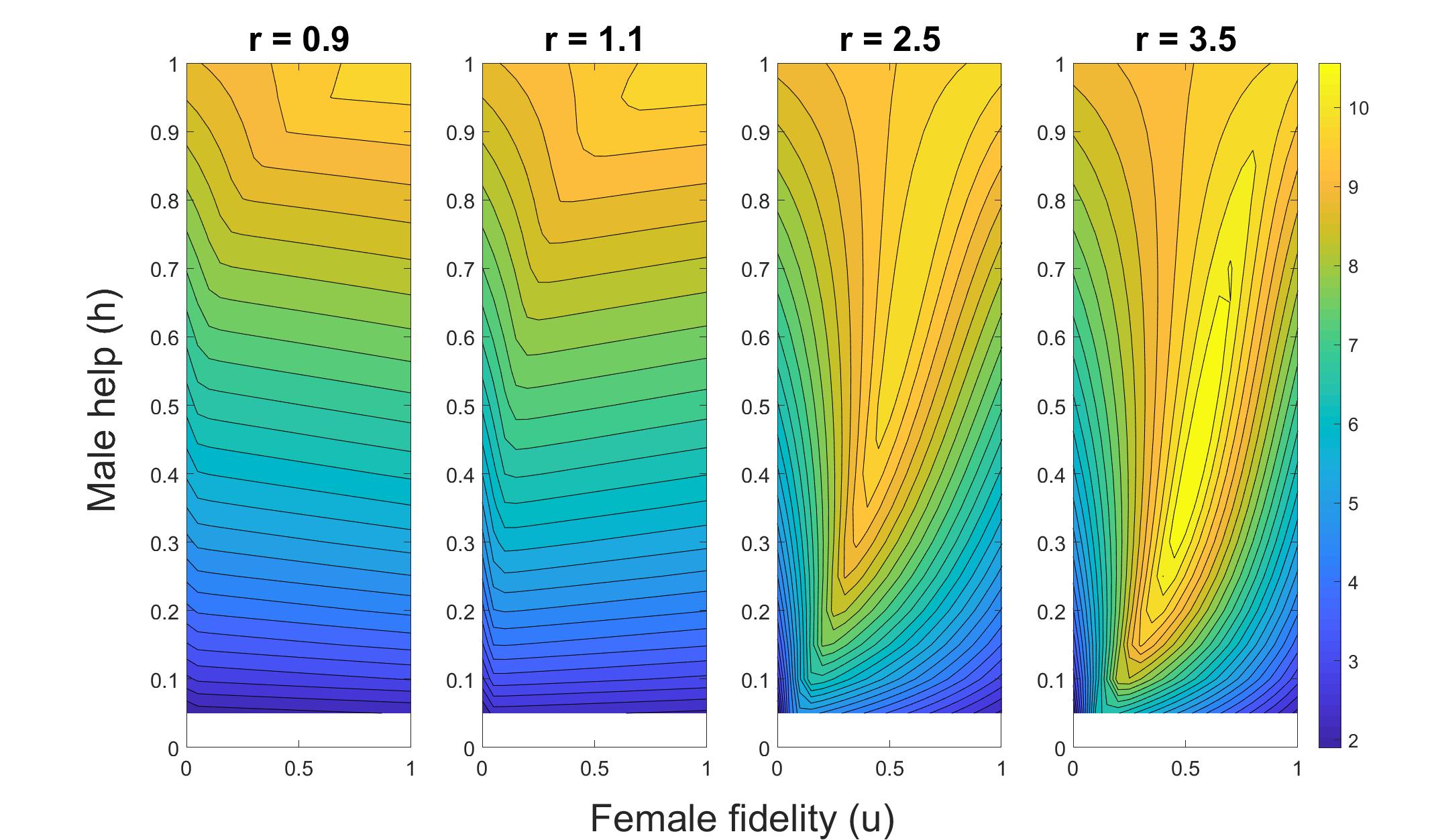


Figure S10. The fitness landscape under the non-overlapping generation model, represented by the per capita growth rate of the population at evolutionary equilibrium, under different $r$ (survival rate of extra-pair offspring relative to their within-pair siblings) at different levels of female fidelity and male help combinations. The efficiency of mate guarding $\delta=0.9$. The empty parameter region at low values or female fidelity and/or male help is due to population extinction caused by low survival rate of the offspring.


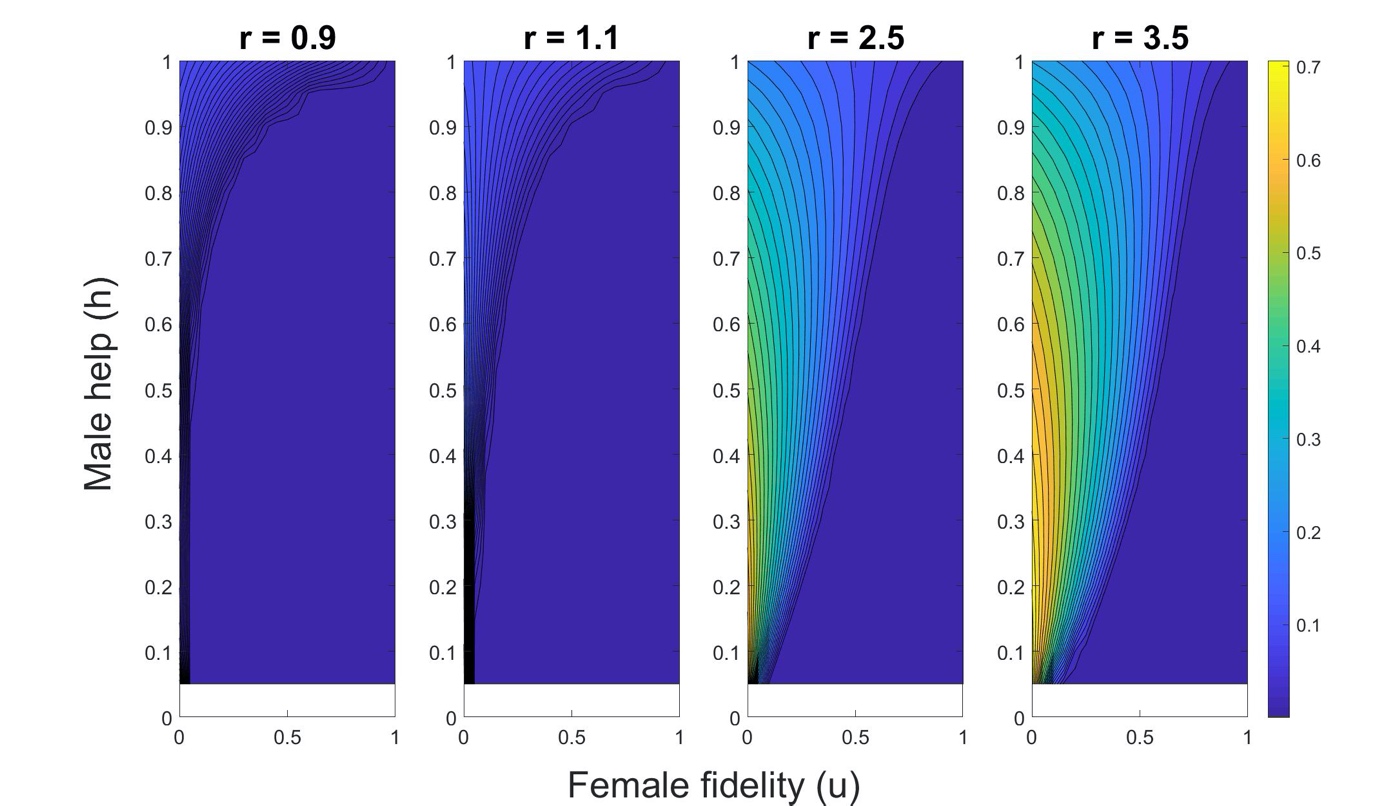


Figure S11. Equilibrium frequency of sneakers in the population under the non-overlapping generation model at different levels of female fidelity and male help combinations. The parameters used for the numerical solutions correspond to Figure S10, with the efficiency of mate guarding $\delta=0.9$.

1. When sneakers are absent in the population and only male help or female fidelity can evolve

Figure S12. Heatmaps of the population states corresponding to Figure 2 of the main text, when the evolution of sneaker frequency was artificially prevented by setting the initial frequency of the $a$ allele to 0 and turning off mutations at this locus. Same as in Figure 2 of the main text, the left column represents the results when male help $h$ is fixed while female fidelity $u$ can evolve, and the right column represents the results when female fidelity $u$ is fixed while male help $h$ can evolve. The data at the same pixel position across the two panels in the same column were generated from the same realization of simulation. Each simulation was run for 5000 generations and the value at each pixel is the mean of the last 500 generations. In each simulation, the relative survival rate of EPO (extra-pair offspring) was 1.1, population size was 5000. The initial degree of male help $h$ was set to 0.5 in the left column, and the initial degree of female fidelity was set to 0.5 in the right column.

1. Additional trajectories of the evolutionary cycles

Figure S13. Evolutionary cycles of the frequency of sneakers and the degrees of male help and female fidelity, when the sneakers have a competitive advantage in extra-pair fertilizations. The trajectories are independent realizations with exactly the same parameter input as in Figure 4a ($\beta=2$).

Figure S14. Evolutionary cycles of the frequency of sneakers and the degrees of male help and female fidelity, when the sneakers have a competitive advantage in extra-pair fertilizations. The trajectories are independent realizations with exactly the same parameter input as in Figure 4b ($\beta=3$).

1. Evolutionary cycles do not occur when sneakers are absent

We explain in this section why the evolutionary cycles we showed in Figure 4 of the main text and the above Figures S13 and S14 do not emerge, when the frequency of sneakers is artificially fixed to zero.


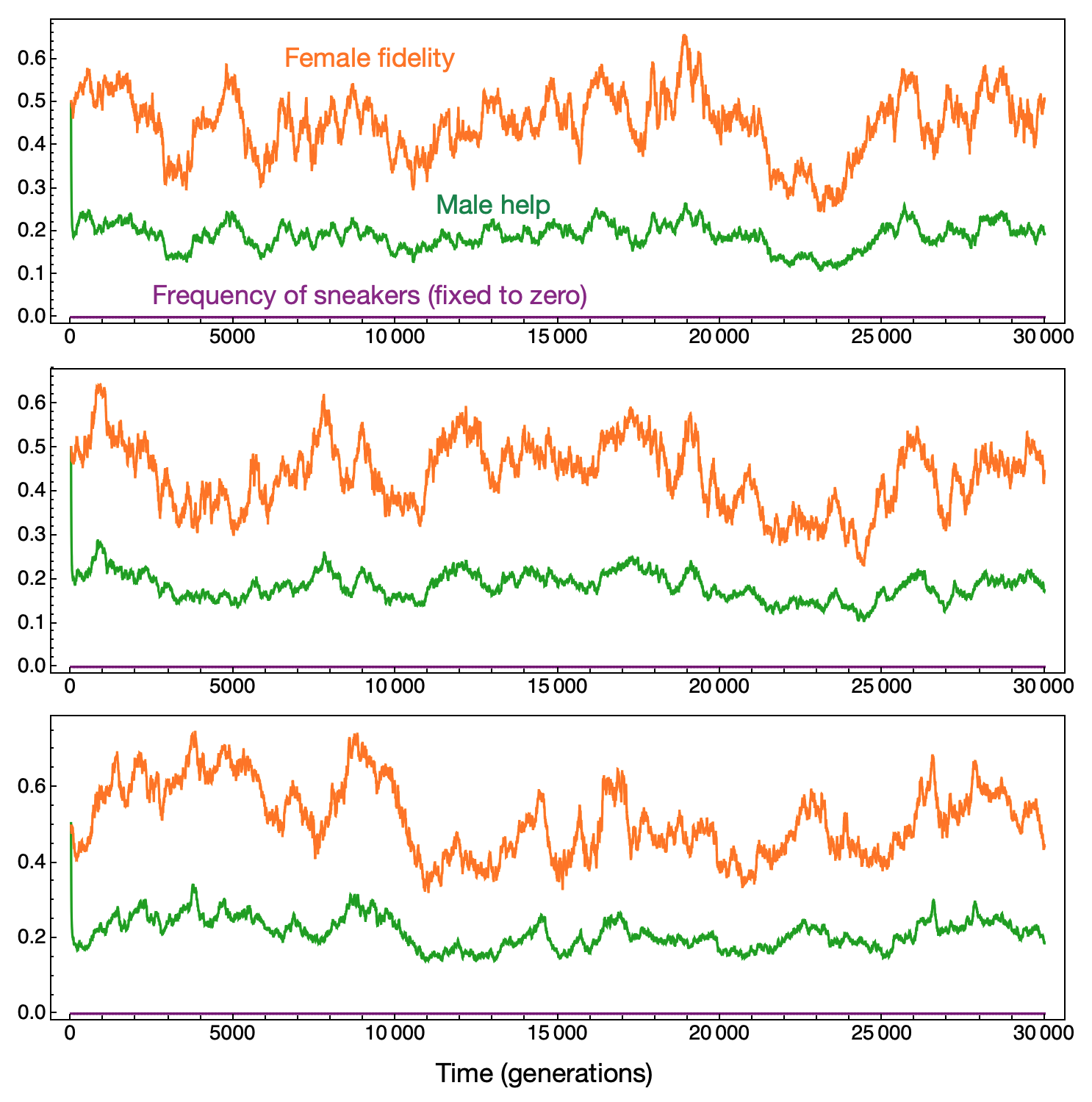


Figure S15. The coevolutionary trajectories of female fidelity and male help when sneakers are absent. When the extra- and within-pair offspring have the same survival rate ($r=1$) and mate guarding is not efficient ($\delta=0$), there is no selection on female fidelity because the within- and extra-pair offspring are equally valuable for the females. Consequently, female fidelity fluctuates around the initial value ($u=0.5$) by random drift. And since mate guarding is not efficient, the bourgeois males cannot actively influence their share of paternity and can only passively respond to the current level of fidelity of females. Therefore, the trajectory of male help follows the same pattern as the female fidelity (the bourgeois males provide relatively more help when female fidelity happens to be relatively high and vice versa, as they balance between gaining within- and extra-pair fertilization opportunities), always at relatively low levels.

To understand why the cycles can emerge when sneakers are present but disappear when there are only bourgeois males in the population, we draw an analogy to two published models in a different coevolutionary context, namely, the coyness game proposed by Dawkins (1989). The model considers a population where females can either be “coy” or “fast” when being courted by a male. The “fast” females accept copulation immediately while the “coy” females insist an extended period of courtship during which they inspect whether the male has the characters of being a good father to their offspring. Correspondingly, there are also two types of males. The “faithful” males are willing to invest in the energetically costly and time-consuming courtship demanded by the “coy” females, while the “philanderer” males move on to search for the “fast” females. After mating, the “faithful” males stay to help the female raise the brood while the “philanderer” males desert their broods. The model assumes that females mate only once.

The model of Schuster and Sigmund (1981) studied the game using a classic evolutionary game theory approach with fixed payoffs for the female and male player under different strategy combinations and indeed, they found the following evolutionary cycles: when the “coy” females are abundant, the “philanderer” males have little chance to mate and resultantly the “faithful” males have higher fitness and increase in frequency; when most males in the population are “faithful”, the “fast” females have an advantage because they save the cost of inspecting males during the extended courtship period and therefore can start reproducing with a (most likely) faithful mate right away, so the frequency of “fast” females increase in the population; this then gives “philanderer” males the opportunity to invade; and when the frequency of “philanderer” males are high in the population, the “coy” females have higher fitness and increase in frequency, leading the cycle back to the beginning.

Later on, McNamara et al. (2008) reexamined the coyness game and pointed out that the assumption of fixed payoff for each sex under different strategy combinations is not realistic, and modified the model to incorporate the frequency-dependent nature of the payoffs. What they found is that under a balanced sex ratio, there are two alternative evolutionarily stable outcomes, namely, either all females are fast and both types of males coexist, or all females are coy and both types of males coexist. Depending on the initial condition, the population evolve to one of the two alternative ESSs and stays there forever. Since one type of female goes extinct at the ESS, there is no possibility for the system to switch between the two evolutionarily stable states. This situation is analogous to what we showed in Figure R1, where without alternative male strategies (the sneakers) the system is trapped to a stable ESS and cannot escape. In contrast, when sneakers are constantly present in the population (by mutations and Mendelian inheritance), the population has the opportunity to switch between different evolutionary optima as the sex-specific fitness landscape evolves [note that, like in McNamara et al. (2008), the payoffs of different male/female strategies are also frequency-dependent in our model]. The periodic switching between two ESSs (a cooperative state where females are of high fidelity and males highly helpful, and a cooperation-breakdown state where females are disloyal and males not helpful) led to the evolutionary cycles.

**References:**

Dawkins, R. 1989. The selfish gene. 40th Anniv. Oxford University Press.

McNamara, J. M., L. Fromhage, Z. Barta, and A. I. Houston. 2008. The optimal coyness game. *Proc. R. Soc. B* **276**:953–960.

Schuster, P., and K. Sigmund. 1981. Coyness, philandering and stable strategies. *Anim. Behav*. **29**:186–192.

1. Sample evolutionary trajectories under coevolution

In this section, we present four sets of coevolutionary trajectories that correspond to four different pixel positions in Figure 3b.


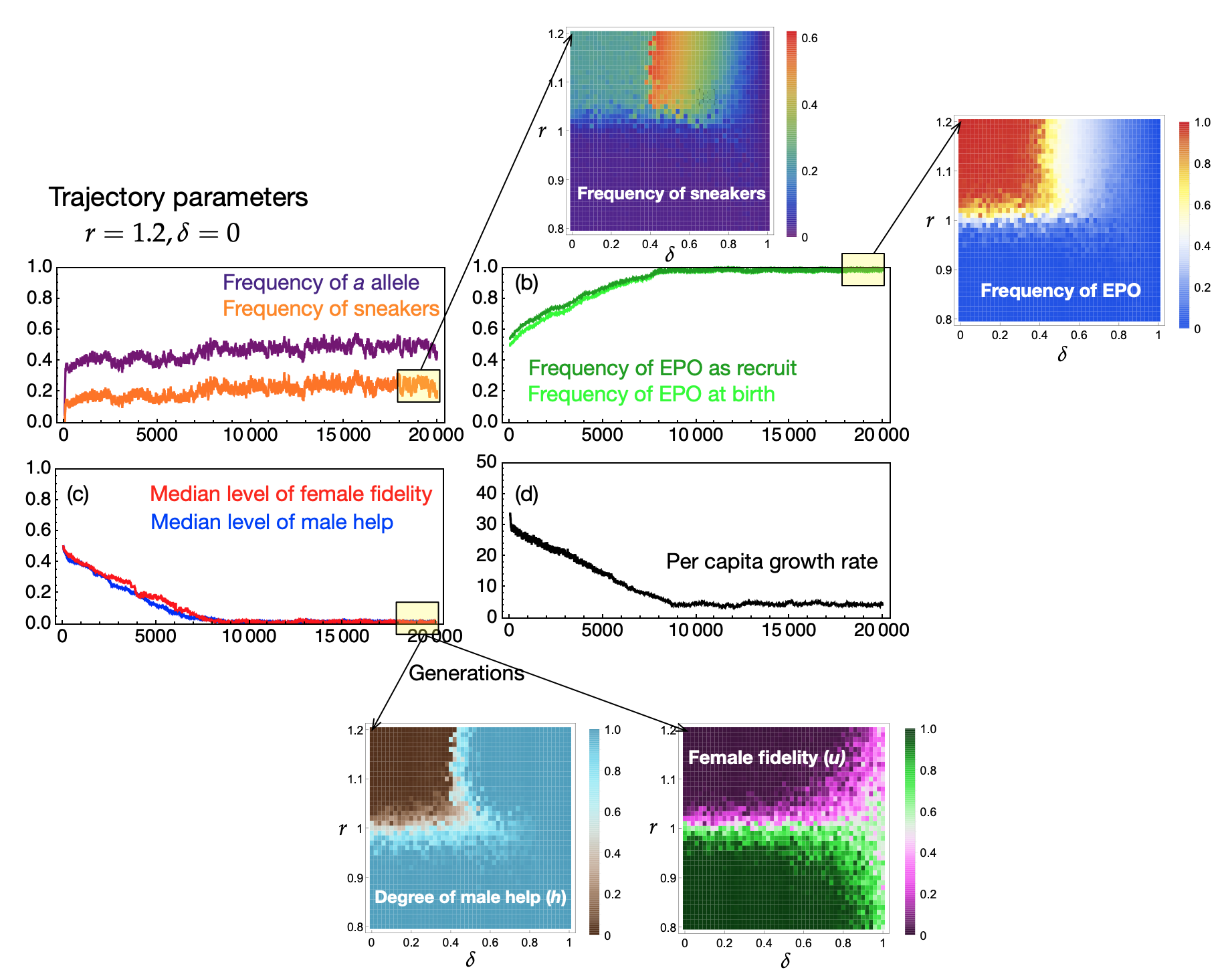


Figure S16. Evolutionary trajectories produced for generating a pixel on the heatmaps of Figure 3b. Here the relative advantage of extra-pair offspring was $r=1.2$, and the efficiency of mate guarding was $\delta=0$. This set of trajectories therefore corresponds to the pixels at the top left corners of the heatmaps in Figure 3b. The mean of the last 2000 generations of the “frequency of sneakers” trajectory in panel (a) was used for the corresponding pixel in panel “Frequency of sneakers” in Figure 3b; The mean of the last 2000 generations of the “frequency of EPO at birth” (the light green trajectory) in panel (b) was used for the panel “Frequency of EPO” in Figure 3b; the information in panel (c) was used for the panels “Degree of male help” and “Female fidelity” in Figure 3b. The per capita growth rate in panel (d) was recorded for diagnostic purposes.


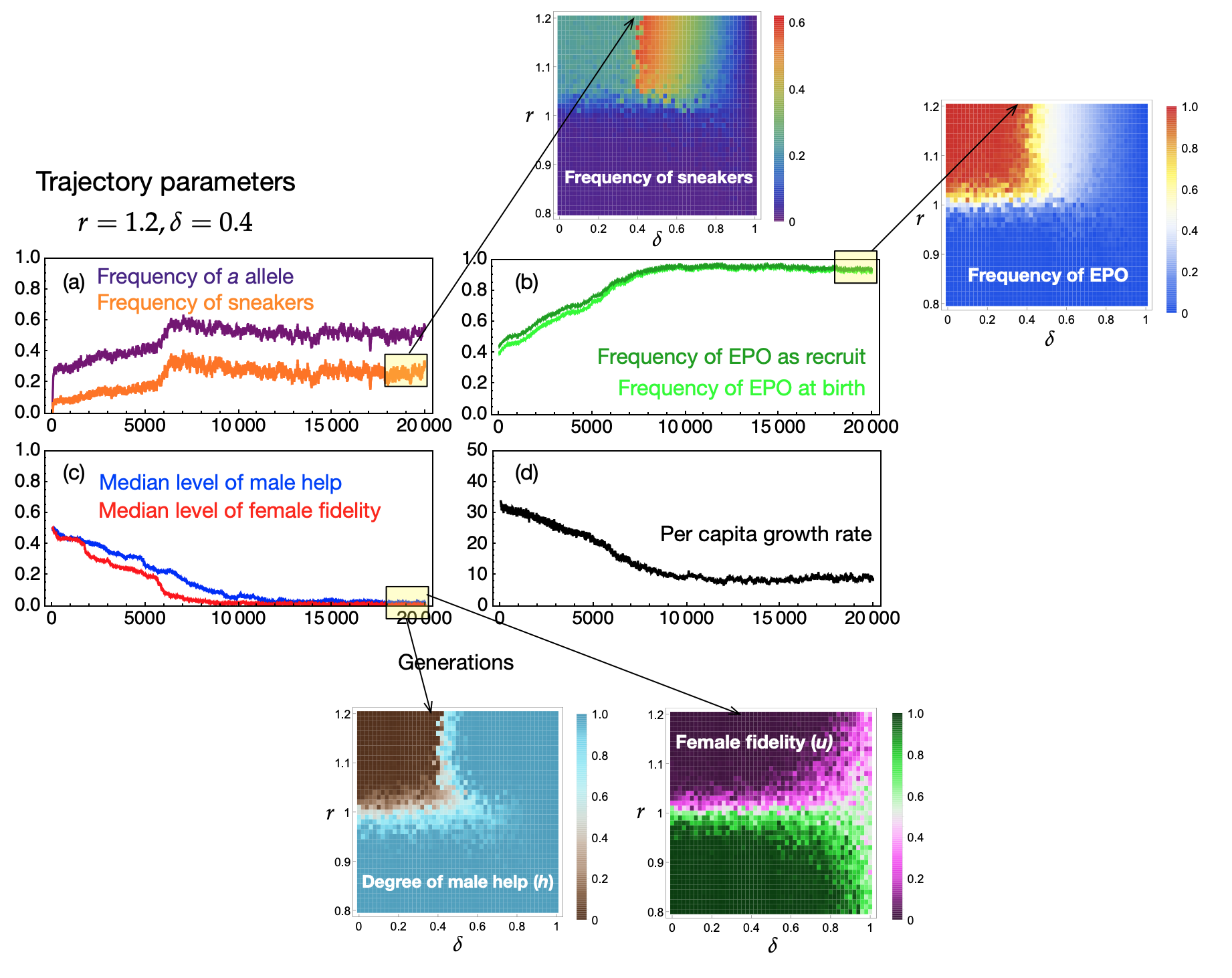


Figure S17. Evolutionary trajectories produced for generating the pixels at $r=1.2$, $\delta=0.4$ in the heatmaps of Figure 3b. The panel arrangements are the same as in Figure S16.


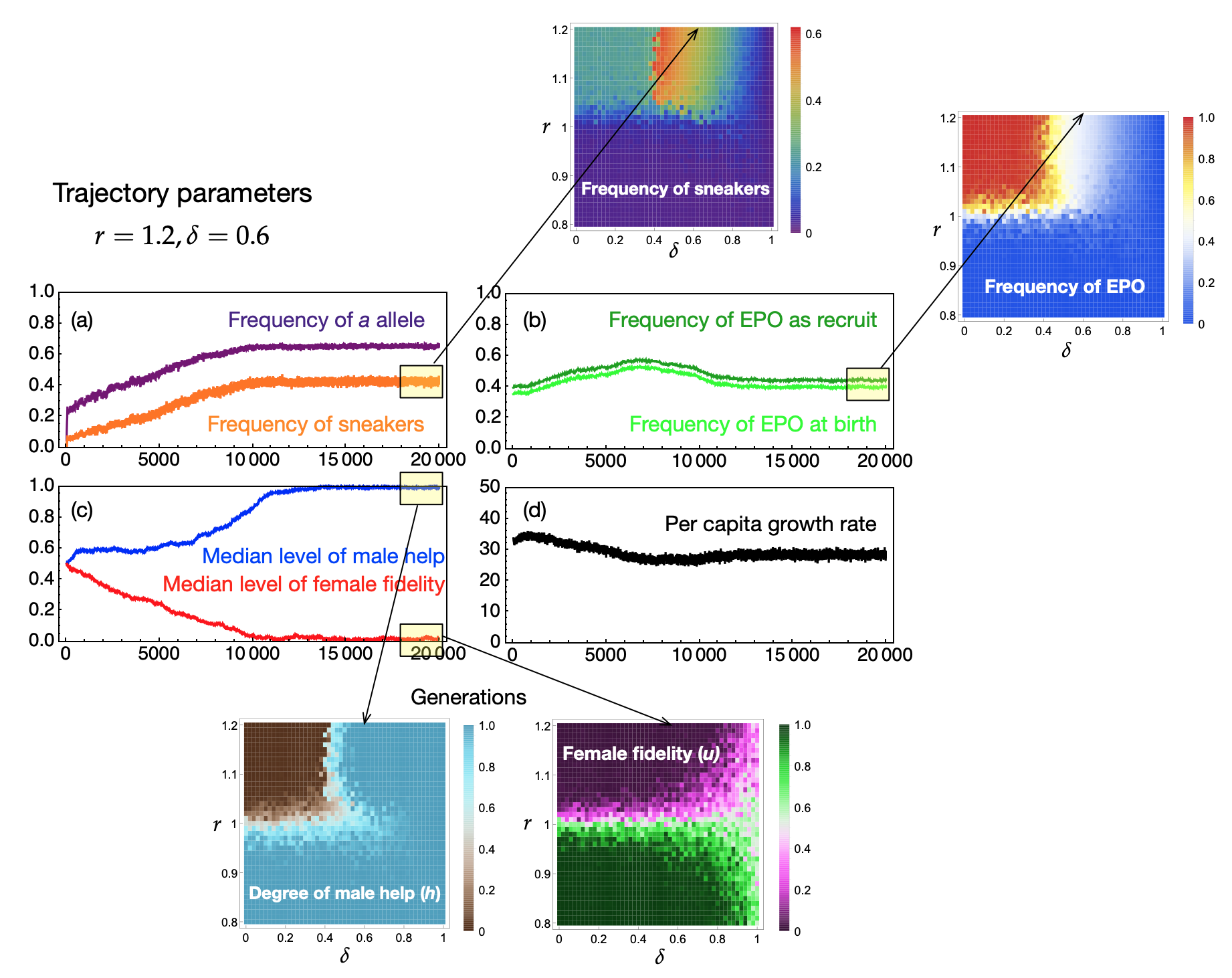


Figure S18. Evolutionary trajectories produced for generating the pixels at the $r=1.2$, $\delta=0.6$ of the heatmaps in Figure 3b. The panel arrangements are the same as in Figure S16.


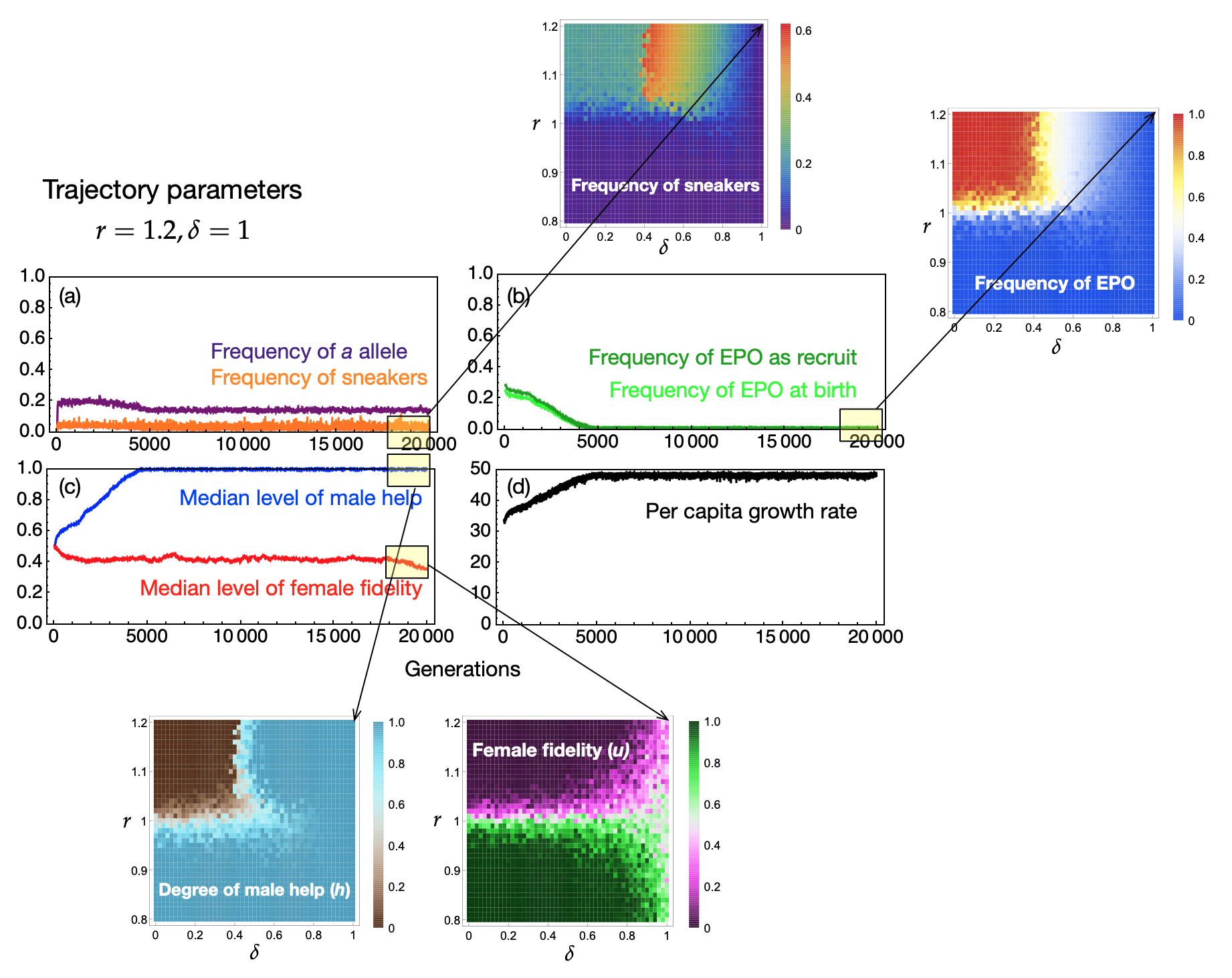


Figure S19. Evolutionary trajectories produced for generating the pixels at the top right corners ($r=1.2$, $\delta=1$) of the heatmaps in Figure 3b. The panel arrangements are the same as in Figure S16.
